# Protein Biochips with Three-Dimensional Hydrogel or Polymer Brush Elements for the Detection of Human Serum Immunoglobulin E Specific to Inhalation Allergens

**DOI:** 10.3390/ijms252313047

**Published:** 2024-12-04

**Authors:** Rinat A. Miftakhov, Georgiy F. Shtylev, Daria A. Kachulyak, Ivan Yu. Shishkin, Vadim A. Vasiliskov, Veronika I. Butvilovskaya, Viktoriya E. Kuznetsova, Valeriy E. Shershov, Victor E. Barsky, Sergey A. Polyakov, Olga A. Zasedateleva, Alexander V. Chudinov

**Affiliations:** Engelhardt Institute of Molecular Biology, Russian Academy of Sciences, 32 Vavilov Street, 119991 Moscow, Russia

**Keywords:** protein microarray, biological microchips, hydrogel or brush elements, UV- or chemically induced immobilization, protein–protein interaction, allergen–sIgE complexes, allergy diagnostics in vitro

## Abstract

The development of rapid analysis of human serum for the presence of allergen-specific Immunoglobulin E (IgE) is currently important. Consequently, we developed two types of three-dimensional (3D) protein biochips. The first one is a 3D hydrogel biochip containing hydrogel droplets with protein molecules (allergens, immunoglobulins and others). These droplets are disposed on elements consisting of short polymer brushes grafting from a surface of polybutylene terephthalate polymer. The immobilization of proteins was induced by short-wave ultraviolet (UV) radiation. On such a biochip, the kinetics of allergen–sIgE complex formation reached 60% of saturation for 6 h. Also, we developed a 3D brush microchip containing on the surface of a polyethylene terephthalate polymer the brush elements with protein molecules covalently immobilized by opening oxirane cycles by amino and thiol nucleophilic groups contained in proteins. In the case of the 3D brush microchip, the kinetics of allergen–sIgE complex formation reached 100% of saturation for 3 h, and fluorescent signals were 2–3 times higher than those of the 3D hydrogel biochip for some allergens. Thus, the comparative analysis revealed that 3D brush biochips are more useful for further studies of protein–protein interaction than 3D hydrogel ones.

## 1. Introduction

Biological microchips (microarrays) with immobilized protein molecules are a rapidly developing field of biotechnology for the creation of medical test systems and for use in basic research [1,2]. The development of rapid diagnostics for socially significant diseases, including the detection of allergic reactions in vitro, is currently an important task. An example of the successful use of biological microchips with immobilized allergen molecules in allergy diagnostics is the technology ImmunoCAP^®^ ISAC [3,4,5].

Immuno Solid-Phase Allergen Chip 112 (ISAC 112) contains 112 allergen components (allergen extracts and recombinant proteins) immobilized on the surface of a glass support [6,7,8]. ISAC 112 allows, having 30 µL of serum or blood plasma, as well as fluorescently labeled anti-human IgE detection antibody, to perform an analysis for sIgE in 4 h [8]. Another example of a biochip is the Allergy Explorer (ALEX) macroarray [9], which contains 282 allergen components (allergen extracts and molecular components) immobilized on the nitrocellulose membrane in a cartridge chip. The analysis using the ALEX macroarray is carried out in approximately 3 h using 100 µL of serum (diluted five times), as well as anti-human IgE antibodies modified with alkaline phosphatase, followed by the addition of an enzyme substrate [6,9]. ISAC and ALEX multiplex arrays were used for comparative analysis of allergy diagnosis [10].

Recently, another study performed by Silimavicius L. et al. has demonstrated the creation of a technology of biochips containing recombinant allergen proteins printed onto 2D epoxy glass slides [11]. The analysis time on such a biochip, using 80 µL of serum (diluted five times) and fluorescently labeled anti-human IgE, was approximately 3 h. In this study, ISAC and ALEX multiplex arrays were used as reference test systems.

When immobilizing protein molecules (allergens, immunoglobulins and other proteins) as probes, it is very important to create conditions in which these molecules would retain their native properties and do not denature during the production and storage of microchips. A suitable medium for proteins is a low-density acrylamide gel [12,13].

Protein microchips, containing protein molecules immobilized in three-dimensional polyacrylamide gel elements on a glass surface, are currently produced only for experimental studies [14,15,16,17]. Unlike protein microchips, biological microchips with DNA molecules immobilized in 3D gel elements on glass or plastic have found wide application for the diagnosis of socially significant diseases, especially for the analysis of resistance of *Mycobacterium tuberculosis* to anti-TB drugs used in the treatment of this disease. In the Russian Federation, a TB biochip containing 77 gel elements has passed the appropriate certification and has been included in the laboratory diagnostic complex [18]. The TB biochip is successfully used in more than 20 anti-tuberculosis clinics. A further development of the TB biochip was the biochip-based TB test system, which is capable of detecting the resistance of *Mycobacterium tuberculosis* to the main drugs used in treating tuberculosis—rifampicin, isoniazid, ethambutol, fluoroquinolones, aminoglycosides and capreomycin [19].

In an alternative method of biochip manufacturing, biomolecules are immobilized to biopolymers in the form of brushes grown on a biochip support using a photomask. This approach has been demonstrated for DNA immobilization and hybridization analysis [20,21,22]. Also, physical immobilization of antibodies in polymer brushes followed by sandwich immunoassay was shown [23]. Covalent immobilization of antibodies in the volume of polymer brushes grafted from or to a semiconductor surface has been developed for biosensing applications [24].

In order to use protein microchips as a diagnostic tool for various socially significant diseases, for example, for allergy diagnostics based on the analysis of immunoglobulins E in patient serum for the specificity of binding to immobilized biochip allergens, it is necessary to improve the available technology in several parameters and to overcome a number of drawbacks.

One such significant drawback is the slow kinetics of the interaction of allergen and other antigen molecules immobilized in the biochip gel elements with specific human serum immunoglobulins (sIgE). In previous studies, the incubation time of allergens and other biochip antigens with blood serum was 20 h, which was necessary for further detection of fluorescent signals from the biochip elements [14,17,25,26]. However, the duration of the immunoassay in a commercial test system should not exceed one working day, or approximately eight hours.

The second significant drawback is associated with the need to switch to the use of a plastic substrate consisting of a polybutylene terephthalate polymer as a biochip substrate, which has been successfully proven to be commercially suitable for the production of DNA microchips [27]. The fact is that such a substrate is produced by high-pressure casting at high temperatures, as a result of which the surface of such a substrate becomes largely inert. The content of chemical groups necessary for reliable attachment of gel elements with protein to such a surface turns out to be negligible.

In our work, we describe a 3D hydrogel microchip containing hydrogel droplets with protein molecules (allergens, immunoglobulins and others) disposed on elements consisting of short polymer brushes grafted from a surface made of polybutylene terephthalate polymer. Protein molecules were immobilized during co-polymerization induced by short-wave UV radiation. On such a biochip, the kinetics of allergen–sIgE complex formation reached 60% of saturation for 6 h, and the immunoassay took 7.5 h. We also offer a 3D brush microchip containing on the surface of a polyethylene terephthalate polymer the brush elements with protein molecules (allergens, immunoglobulins and others) covalently immobilized by opening oxirane cycles with nucleophilic groups contained in the protein (amino and thiol groups). In the case of 3D brush microchips, the kinetics of allergen–sIgE complex formation reached 100% of saturation for 3 h, and the immunoassay was performed for 5 h.

## 2. Results

### 2.1. Immunofluorescence Analysis on a 3D Hydrogel Biochip

**Figure 1:**
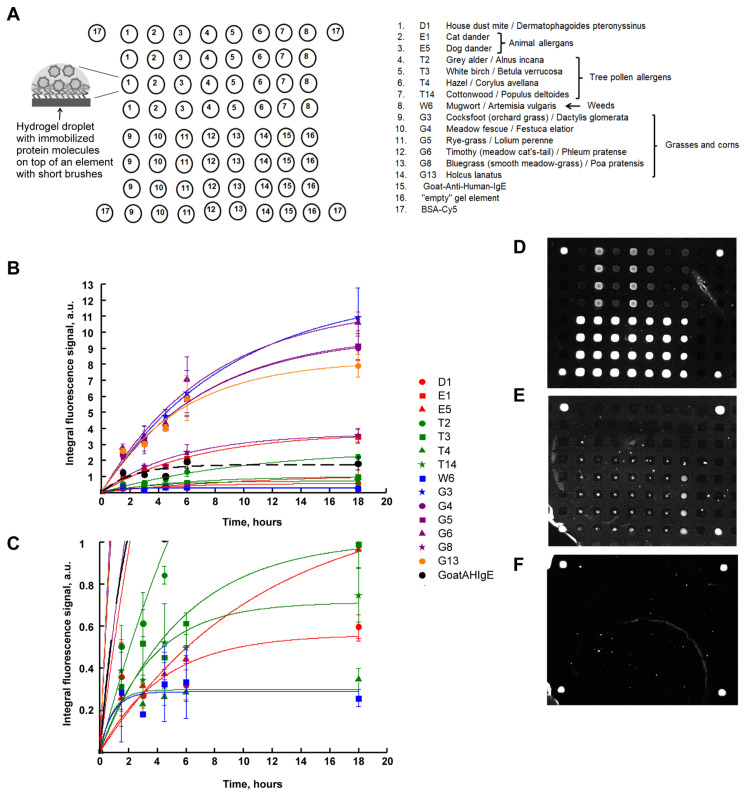
Immunofluorescence analysis on a 3D hydrogel biochip. (**A**) Scheme of the biochip. (**B**) Kinetic curves of the interaction of allergens with sIgE of human blood serum. The value of the fluorescent signal for each allergen (or antibody) is averaged over 2 chips (over 8 gel elements) and is marked by a symbol. The bars indicate the absolute deviations. Curves indicate the approximation of the experimental values marked by symbols using the least squares method according to the following exponential equation [28]: y = A × (1 − exp(−x/B)), where A is maximal value of y (fluorescent signal), x is the time after the addition of human blood serum to the biochip, and B is a parameter equal to the time at which allergen–sIgE complex formation reached 63% of saturation. The approximation curves for elements with allergens are shown as colored solid lines, and for elements with an antibody, a black dashed line is shown. (**C**) The plot in (**B**) shown at the increased y-axis scale at low values of fluorescence signals. (**D**) An image of a 3D hydrogel biochip after 6 h incubation with a control positive (inhalation) serum (CS+) and the development of the obtained allergen–sIgE complexes with Goat Anti-Human-IgE-Cy5 antibodies. (**E**) Image of a 3D hydrogel biochip after 6 h incubation with a control negative serum (CS−) and development of the obtained complexes formed between Goat-Anti-Human-IgE antibodies and IgE with Goat Anti-Human-IgE-Cy5 antibodies. (**F**) Image of a 3D hydrogel biochip after 6 h incubation with a zero-calibration sample (CS0) followed by 1 h incubation with Goat Anti-Human-IgE-Cy5 antibodies.

Figure 1A shows a scheme of the developed 3D hydrogel biochip. To manufacture such a biochip, first, photolithography is performed, where a matrix of elements in the form of short polymer brushes consisting of polyethelene glycol methacrylate was obtained. Polymer brushes were covalently fixed at one end on the surface of polybutylene terephthalate plastic [21,22]. Then, using a robot with a pin, an aqueous methacrylamide–glycerin–salt solution of the protein was applied, with the addition of TEMED as a polymerization catalyst for the gel elements of the biochip. The immobilization of biomolecules occurred in the process of co-polymerization induced by short-wave UV radiation with a maximum at a wavelength of 312 nm.

The images were recorded with an exposure of 1 s in the fluorescence range of Cy5 dye. As Figure 1A shows, the biochip contained 14 immobilized allergens: house dust mite D1, animal allergens E1 (cat dander), E5 (dog dander), tree pollen allergens T2 (gray alder), T3 (white birch), T4 (hazel), T14 (cottonwood), weeds W6 (mugwort), grasses and corns G3 (cocksfoot), G4 (meadow fescue), G5 (rye-grass), G6 (timothy), G8 (bluegrass) and G13 (holcus lanatus), each of which was immobilized in four repeats. Also, the biochip contained goat polyclonal antibodies to human IgE (Goat Anti-Human-IgE), “empty” gel elements and the protein BSA-Cy5 as a marker.

Figure 1B shows the kinetic curves of the interaction of allergens with specific IgE of human blood serum. Figure 1C shows the kinetic curves which are at low values of fluorescence signals. As one can see, according to the kinetic curves (see also Table 1), the time of saturation of the fluorescent signals differed for the allergens, but on average, it was 22 h. The time for the fluorescent signal to reach 60% from saturation was approximately 6 h.

Also, in Figure 1B, a broken line shows the kinetic curve of the interaction of immobilized specific IgG (Goat Anti-Human-IgE) with serum IgE. As can be seen from the figure, the saturation time for the formation of corresponding complexes from antibodies was 9 h (see also Table 1).

Figure 1D–F show images of hydrogel biochips after 6 h incubation with a control positive (inhalation) serum (CS+) (Figure 1D), a control negative serum (CS−) (Figure 1E) or with a zero-calibration sample (CS0) (Figure 1F), each followed by a 1 h incubation in the presence of Goat Anti-Human-IgE-Cy5.

In the case of incubation with CS+, it is seen that elements containing allergens and Goat Anti-Human-IgE fluoresced with different intensities. Thus, CS+ contains sIgE to biochip allergens.

In the case of incubation with CS−, the gel elements of the biochip containing immobilized Goat Anti-Human-IgE antibodies fluoresced (Figure 1E), which is consistent with the composition of CS−.

In the case of CS0, only marker elements containing immobilized BSA–Cy5 fluoresced (Figure 1F), which is consistent with the composition of CS0.

It should also be noted that in all images of biochips in Figure 1D–F, there are no signals from “empty” gel elements.

### 2.2. Immunofluorescence Analysis on a 3D Brush Biochip

**Figure 2:**
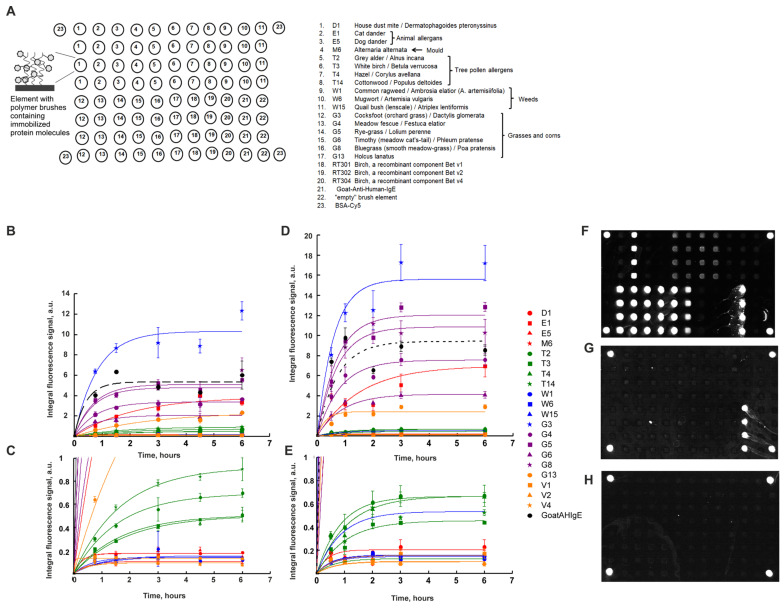
Immunofluorescence analysis on a 3D brush biochip. (**A**) Scheme of the biochip. (**B**) Kinetic curves of the interaction of allergens with sIgE of human blood serum. The value of the fluorescent signal for each allergen (or antibody) was averaged over 1 chip (over 4 gel elements) and is marked by a symbol. The bars indicate the absolute deviations. Curves indicate the approximation of the experimental values marked by symbols using the least squares method as described in the legend to Figure 1B. The approximation curves for elements with allergens are shown as colored solid lines, and for elements with an antibody, a black dashed line is shown. (**C**) The plot in (**B**) shown at the increased y-axis scale at low values of fluorescence signals. (**D**) Kinetic curves of the interaction of allergens with sIgE of human blood serum obtained with mixing. (**E**) The plot in (**D**) shown at the increased y-axis scale at low values of fluorescence signals. (**F**) An image of a 3D brush biochip after 3 h incubation with a control positive (inhalation) serum (CS+) with mixing and the development of the obtained allergen–sIgE complexes with Goat Anti-Human-IgE-Cy5 antibodies. (**G**) Image of a 3D brush biochip after 3 h incubation with control negative serum (CS−) with mixing and development of the obtained complexes formed between Goat-Anti-Human-IgE antibodies and IgE with Goat Anti-Human-IgE-Cy5 antibodies. (**H**) Image of a 3D brush biochip after 3 h incubation with a zero-calibration sample (CS0) with mixing followed by 1 h incubation with Goat Anti-Human-IgE-Cy5 antibodies. The images were recorded with an exposure of 1 s in the fluorescence range of Cy5 dye.

Figure 2A shows a scheme of the developed 3D brush biochip. To manufacture such a biochip, first, photolithography is performed, where a matrix of polymers in the form of brushes was made from monomers of glycidyl methacrylate. Epoxy groups are able to react with amino and thiol groups in the protein without additional activation. Then, using a robot with a pin, an aqueous glycerin protein solution was applied, also containing triethylamine, as a catalyst for the opening of the oxirane cycle.

As Figure 2A shows, the biochip contained 20 immobilized allergens:

house dust mite D1,

animal allergens E1 (cat dander), E5 (dog dander),

mold M6 (Alternaria alternate),

tree pollen allergens T2 (gray alder), T3 (white birch), T4 (hazel), T14 (cottonwood),

weeds W1 (common ragweed), W6 (mugwort), W15 (quail bush (lenscale)),

grasses and corns G3 (cocksfoot), G4 (meadow fescue), G5 (rye-grass), G6 (timothy), G8 (bluegrass) and G13 (holcus lanatus),

recombinant components of birch Bet v1, Bet v2 and Bet v4,

each of which was immobilized in four repeats. Also, the biochip contained goat polyclonal antibodies to human IgE (Goat Anti-Human-IgE), “empty” gel elements and the protein BSA-Cy5 as a marker.

Figure 2B,C show the kinetic curves of the interaction of allergens with specific IgE of human blood serum. As one can see, according to the kinetic curves, the time of saturation of the fluorescent signals varied from 0.2–2 (for D1, E5, M6, Bet V1 and Bet V4) to 8–11 h (for E1 and G13). The data are summarized in Table 1. The time of saturation averaged for all allergens was 4 h.

Figure 2D,E show the kinetic curves of the interaction of allergens with specific IgE of human blood serum obtained with mixing. As one can see, according to the kinetic curves, the time of saturation of the fluorescent signals shortened to 3 h on average for all allergens (in comparison with the time of saturation obtained without mixing; see Table 1). At the same time, the fluorescent saturation signals increased 1.5 times for some allergens.

Figure 2F–H show images of hydrogel biochips after 3 h incubation with a control positive (inhalation) serum (CS+) with mixing (Figure 2F), a control negative serum (CS−) with mixing (Figure 2G) or with a zero-calibration sample (CS0) with mixing (Figure 2H), as well as a 1 h incubation in the presence of Goat Anti-Human-IgE-Cy5.

In the case of incubation with CS+, it is seen that elements containing allergens and Goat Anti-Human-IgE fluoresced with different intensities. Thus, the biochip revealed sIgE in CS+.

In the case of incubation with CS−, the gel elements of the biochip containing immobilized Goat Anti-Human-IgE antibodies fluoresced (Figure 2G), which is consistent with the composition of CS−.

In the case of CS0, only marker elements containing immobilized BSA–Cy5 fluoresced (Figure 2H), which is consistent with the composition of CS0.

It should also be noted that in all images of biochips in Figure 2D–F, there are no signals from “empty” gel elements.

### 2.3. Analysis of Fluorescent Signals on 3D Hydrogel and 3D Brush Biochips, Comparison with ELISA Results

**Figure 3:**
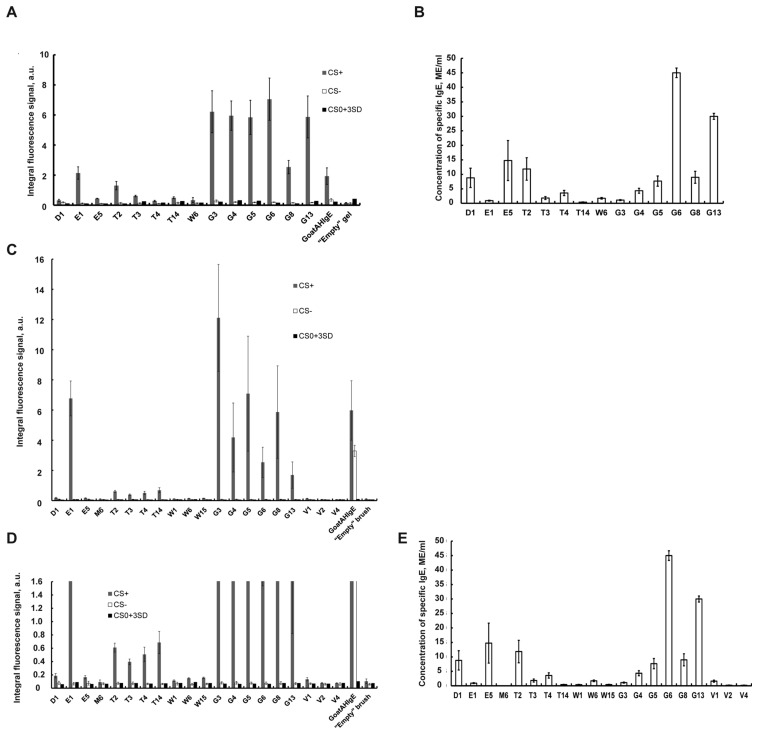
Analysis of fluorescent signals on 3D hydrogel and 3D brush biochips, and comparison with ELISA results. (**A**) Histogram of the averaged fluorescent signals obtained from 3D hydrogel biochips for gel elements with immobilized 14 allergens, goat polyclonal antibodies and an “empty” gel in the case of incubation for 6 h with a control positive inhalation, CS+ (gray columns), with a control negative serum, CS− (white columns) and with a zero-calibration sample CS0 (black columns). The value of the fluorescent signal for each allergen (or antibody) is averaged over 2 biochips (over 8 gel elements). The bars indicate the absolute deviations. (**B**) Histogram of the concentrations of sIgE in CS+ determined by enzyme immunoassay (ELISA) for the same 14 allergens as in (**A**). The bars indicate the measurement errors. (**C**) Histogram of the averaged fluorescent signals obtained from 3D brush biochips for brush elements with immobilized 20 allergens, goat polyclonal antibodies and an “empty” gel in the case of incubation for 3 h with a control positive inhalation, CS+ (gray columns), with a control negative serum, CS− (white columns) and with a zero-calibration sample CS0 (black columns) with mixing. The value of the fluorescent signal for each allergen (or antibody) is averaged over 2 biochips (over 8 gel elements). The bars indicate the absolute deviations. (**D**) The histogram in (**C**) shown at the increased y-axis scale at low values of fluorescence signals. (**E**) Histogram of the concentrations of sIgE in CS+ determined by ELISA for the same 20 allergens as in (**C**,**D**). The bars indicate the measurement errors.

Figure 3A shows a comparative histogram of the averaged fluorescent signals of gel elements with immobilized allergens, goat polyclonal antibodies and an “empty” gel in the case of incubation for 6 h with a control positive inhalation, CS+ (gray columns), with a control negative serum (CS−) (white columns) and with a zero-calibration sample CS0 (black columns) for each allergen of the 3D hydrogel biochip. As can be seen from the histogram, the values of fluorescent signals for gel elements with 14 biochip allergens after incubation with CS+ exceed the values of fluorescent signals in the same gel elements after incubation with CS− or with CS0, as well as the values of signals from “empty” gel elements. Consequently, CS+ contains sIgE to all biochip allergens.

Figure 3B shows a histogram of the concentrations of sIgE in CS+ determined by enzyme immunoassay (ELISA) for the same 14 allergens. As can be seen from the histogram, the sIgE concentration values for all 14 allergens exceeded the value of 0.35 IU/mL. Thus, the ELISA method confirmed the result obtained using a 3D hydrogel biochip, namely the presence of sIgE in CS+ to all 14 allergens considered.

Thus, the analysis of human blood serum using a hydrogel biochip (in which the fluorescent signal of the biochip gel elements reaches 60 percent saturation in 6 h of serum incubation) and the ELISA analysis gave the same qualitative result on the presence of antibodies in the control positive serum to 14 allergens. Therefore, achieving 60% saturation of the fluorescent signals in the elements of the biochip is sufficient to determine the presence of sIgE in the serum.

Taking into account the 1 h development of allergen–sIgE complexes using Goat Anti-Human-IgE-Cy5 antibody, the total time for the analysis of blood serum for sIgE to allergens using a hydrogel biochip is 7.5 h. At the same time, the amount of serum required for the analysis of 14 allergens on a biochip (100 µL) is seven times less than for the analysis of 14 allergens by the ELISA method (50 µL × 14 = 700 µL).

Figure 3C shows a histogram of the averaged fluorescent signals of brush elements with immobilized allergens, goat polyclonal antibodies and an “empty” gel in the case of incubation for 3 h with a control positive inhalation, CS+ (gray columns), with a control negative serum, CS− (white columns) and with a zero-calibration sample CS0 (black columns) with mixing for each allergen of the 3D brush biochip. Additionally, Figure 3D shows the increased y-axis scale at low values of fluorescence signals. As can be seen from the histogram, the values of fluorescent signals for gel elements with 17 biochip allergens after incubation with CS+ exceed the values of fluorescent signals in the same gel elements after incubation with CS− or with CS0, as well as the values of signals from “empty” gel elements. Consequently, CS+ contains sIgE to 17 biochip allergens.

Meanwhile, fluorescence signals from brush elements with three allergens, M6, Bet v2 and Bet v4, do not exceed the values of fluorescent signals in the same gel elements after incubation with CS− or with CS0, as well as the values of signals from “empty” gel elements (Figure 3D). Thus, CS+ does not contain sIgE to allergens M6, Bet v2 and Bet v4.

Figure 3E shows a histogram of the concentrations of sIgE in CS+ determined by enzyme immunoassay (ELISA) for the same 20 allergens. As can be seen from the histogram, the sIgE concentration values for 17 allergens exceeded the value of 0.35 IU/mL. Meanwhile, the concentrations of three allergens, M6, Bet v2 and Bet v4, are lower than 0.35 IU/mL. Thus, the ELISA method confirmed the result obtained using 3D brush biochip.

## 3. Discussion

Allergic diseases are among the most common diseases in the world. The correct diagnosis and the choice of a course of treatment mostly depend on the results of diagnostics. In vivo and in vitro methods are used in the diagnosis of allergies [7,29,30]. Skin prick tests, patch tests, intradermal tests are in vivo diagnostics, the most reliable and frequently used methods in research to confirm allergic reactions. Laboratory methods (in vitro, ELISA and multiplex arrays) complement clinical ones, and in some cases, can be used at the initial stage of allergy diagnosis.

The advantages of using in vitro tests include the following: there are no age restrictions; they can be used for patients with a high degree of sensitization (systemic allergic reactions during skin testing); they are applicable if it is impossible to discontinue antihistamines; and they can be performed during an exacerbation of the underlying disease. However, in vitro tests have a disadvantage in that the detection of a specific IgE (sIgE) to any allergen does not prove that this particular allergen is responsible for clinical symptoms such as asthma or skin itching [30].

In this work, two possibilities for producing biochips (microarrays) with immobilized molecules of allergens, antibodies and other proteins were demonstrated. In the case of 3D hydrogel biochips, proteins are immobilized in the environment of acrylamide gel elements under short-wave UV irradiation. This variant of biochip manufacturing is similar to the previously used technique [12,13,14,15,16,17,25,26], but has a number of changes. If previously hydrogel droplets were attached to the surface of glass, then in our technique, hydrogel droplets with protein are strongly attached to short brushes “grown” on the surface of plastic made of polybutylene terephthalate polymer.

The second difference is the shorter wavelength of irradiation during polymerization of gel cells with protein. Compared to earlier studies [12,13,14,15,16,17,25,26], where a wavelength of 350 nm was used, in our work, gel elements were irradiated at a wavelength of 312 nm, which contributed to a more optimal polymerization of hydrogel elements and allowed us to reach 60% of saturation for 6 h (Figure 1B,C).

In the second variant of manufacturing a protein biochip, allergens, antibodies and other proteins are immobilized in the volume of acrylamide brushes due to the formation of covalent bonds in one-step immobilization of protein molecules, greatly simplifying the manufacturing stages. It should be noted that under UV irradiation used in 3D hydrogel biochips manufacturing, there is a risk of partial denaturation of proteins, which can affect their structural integrity and activity. Compared with 3D hydrogel biochips, protein macromolecules of 3D brush biochips are immobilized in more gentle conditions, without exposure to short-wave radiation.

Also, brush polymers made it possible to achieve 2–3 times higher fluorescent signals in the elements of 3D brush biochips than in the elements of 3D hydrogel biochips.

Another significant advantage of 3D brush biochips is the faster kinetics of the interaction of biomolecules in the brush elements of the biochip. In the brush elements, the fluorescent saturation signal is achieved with mixing in 3 h (Figure 2D,E, Table 1), while in hydrogel elements, the saturation time of the fluorescent signal is 22 h (Figure 1B, Table 1). With mixing, the fluorescence signals increase by 1.5 times for some allergens. The growth of signals and decrease in saturation time in the brush elements during mixing apparently occurs due to a more optimal penetration of biomolecules of the solution to the immobilized probes.

Thus, our work demonstrates an updated version for the manufacture of a 3D hydrogel protein biochip, and a new variant for the manufacture of a 3D brush protein biochip. The possibility of conducting immunofluorescence analysis on these biochips has also been demonstrated. The results obtained on 3D hydrogel and brush biochips concerning the detection of sIgE to allergens qualitatively coincide with the results obtained by the ELISA method. It is shown that the 3D brush biochip is the most optimal, since the saturation time of the fluorescent signal in the brush elements is reduced to 3 h while the fluorescent signals are more than two times higher. Of course, the developed method using 3D brush biochips (taking about 5 h) is longer in time than ELISA (about 3 h), but is apparently less time-consuming and undoubtedly less expensive in terms of serum. The amount of serum required for the analysis of 20 allergens on a biochip (100 µL) is ten times less than for the analysis of 20 allergens by the ELISA method (50 µL × 20 = 1000 µL).

It can be concluded that 3D brush biochips are more suitable as the instrument for further in vitro analysis and studies of protein–protein and other biomolecular interactions.

## 4. Materials and Methods

### 4.1. Manufacture of 3D Hydrogel Protein Biochips

Polymer brushes of polyethylene glycol methacrylate were grafted from a plastic substrate made of polybutylene terephthalate polymer by UV-induced (λ = 254 nm) photolithography using a quartz photomask.

To the resulting matrix of brush elements (200 × 200 microns) using a robot QArray 2 (Genetix, London, UK), the solutions of allergens D1, E1, E5, T2, T3, T4, T14, W6, G3, G4, G5, G6, G8 or G13, (0.4–3 mg/mL, Greer Laboratories, Lenoir, NC, USA) or polyclonal antibodies to human IgE (0.3 mg/mL, Bethyl Laboratories, Montgomery, TX, USA) in the volume of an acrylamide gel with the addition of glycerin and TEMED (3.7% methacrylamide, 0.11% N,N′-methylenebisacrylamide, 50% glycerin, 3.6% TEMED) were applied. Similar solutions containing BSA-Cy5 (0.3 mg/mL, Sigma, St. Louis, MO, USA) were applied in the corners of the biochip; see the scheme of the biochip in Figure 1A.

Next, the biochips were irradiated at a wavelength of 312 nm using a Vilber UV-B T-15M lamp (Vilber, Webster, TX, USA) for 20 min in a nitrogen atmosphere at 22 °C. Then, the biochips were washed in the PBST buffer (0.1% Tween, 150 mM NaCl, 10mM Na–Phosphate buffer, pH 7.2) for 20 min with stirring at 150 r.p.m. at room temperature. The biochips were washed with distilled water and dried in an air stream. After these procedures, the biochips were ready for use.

### 4.2. Conducting Immunoassay on 3D Hydrogel Biochips

On 3D hydrogel biochips in individual chambers, 100 µL of a solution of control positive inhalation serum (CS+, Alkorbio, St. Petersburg, Russia) was applied. On other 3D hydrogel biochips in individual chambers, 100 µL of a solution of control negative serum (CS−, Alkorbio, Russia) or 100 µL of a zero-calibration sample (CS0, Hema, Moscow, Russia) was applied.

The biochips were incubated with CS+ for 1.5, 3, 4.5, 6 and 18 h at 37 °C. The biochips were incubated with CS− and CS0 for 6 h at 37 °C. Then, the biochips were washed with distilled water and dried in an air stream.

Next, the biochips were incubated in 100 µL of polyclonal goat antibodies on human IgE (Bethyl Laboratories, USA), dissolved in PBS buffer (150 mM NaCl, 10 mM Na–Phosphate buffer, pH 7.2) at 0.01 mg/mL. Preliminary goat antibodies were labeled with Cy5 fluorescent dye according to the procedure described earlier [31]. The biochips were incubated in individual chambers at 37 °C for 1 h.

At the next stage, individual chambers were removed from the biochips; they were rinsed in distilled water and placed in the tubes containing 25 mL of a PBST solution diluted 8 times with water, and incubated in this solution for 20 min with stirring at 150 r.p.m., thus destroying non-specific interactions of the goat antibody.

Then, the biochips were washed with distilled water and dried in an air stream.

### 4.3. Manufacturing of 3D Brush Protein Biochips

Elements of 3D brush biochips consisted of brush polymers with active epoxy groups on the plastic surface and were obtained on a plastic substrate made of polybutylene terephthalate polymer by UV-induced (λ = 254 nm) photolithography using a quartz photomask with 200 × 200 microns holes and monomers based on glycidylmethacrylate.

Aqueous solutions of allergens D1, E1, E5, M6, T2, T3, T4, T14, W1, W6, W15, G3, G4, G5, G6, G8, G13, Bet v1, Bet v2 and Bet v4 (3 mg/mL, Alkorbio, Russia) or polyclonal goat antibodies to human IgE (0.3 mg/mL, Bethyl Laboratories, USA) with the addition of glycerin and triethylamine (TEA) (30% of glycerin, 2% TEA) were applied to the resulting matrix of brush elements using a robot QArray 2 (Genetix, UK). Similar solutions containing BSA-Cy5 (0.3 mg/mL, Sigma, USA) were applied in the corners of the biochip; see the biochip scheme shown in Figure 2A.

After immobilization, the biochips were washed with a stream of deionized water and washed in a PBST buffer for 20 min with stirring at a speed of 150 r.p.m. at room temperature. The biochips were washed with distilled water and dried in an air stream. After these procedures, the biochips were ready for use.

### 4.4. Conducting Immunoassay on 3D Brush Biochips

For immunoassay, 100 µL of control positive inhalation serum (CS+, Alkorbio, Russia) was applied to the biochip chambers. In order to evaluate the binding kinetics, 3D brush biochips were incubated with a control positive serum at a temperature of 37 °C for 45 min, 1.5, 3, 4.5, and 6 h both without stirring and with stirring at a speed of 300 rpm.

As a control, brush biochips were incubated with 100 µL of a control negative serum solution (CS−, Alkorbio, Russia) or 100 µL of a zero-calibration sample (CS0, Hema, Russia) for 3 h.

After incubation, the biochips were washed with distilled water and dried in an air stream. Next, the biochips were incubated with polyclonal goat antibodies and further treated as described for 3D hydrogel biochips.

### 4.5. Recording of Fluorescent Images of Biochips

Biochips were recorded in the fluorescence range of Cy5 dye (λ_abs_^max^ = 648 nm, λ_em_^max^ = 670 nm) using a fluorescent LED microscope equipped with a CCD camera and software (IMB RAS, Moscow, Russia). Further, the fluorescent images of the biochips were analyzed using the Image Express program (IMB RAS, Moscow, Russia), which made it possible to calculate the total fluorescent signal (integral fluorescent signal) for each element of the biochip.

### 4.6. CS+ Analysis by Enzyme Immunoassay (ELISA)

Concentrations of IgE specific to allergens D1, E1, E5, M6, T2, T3, T4, T14, W1, W6, W15, G3, G4, G5, G6, G8, G13, Bet v1, Bet v2 and Bet v4 in the control positive inhalation serum, CS+, were determined by the ELISA method using the Alkor bio ELISA kit (Alkor Bio, Russia).

## 5. Conclusions

Our work demonstrates an updated version of the manufacture of a 3D hydrogel protein biochip, and a new variant for the manufacture of a 3D brush protein biochip. The results obtained using 3D hydrogel and brush biochips concerning the detection of sIgE to allergens qualitatively coincide with the results obtained by the ELISA method. It is shown that the 3D brush biochip is the most optimal, since the saturation time of the fluorescent signal in the brush elements is reduced to 3 h while the fluorescent signals are more than two times higher for some allergens.

It can be concluded that 3D brush biochips are more suitable as the instrument for further analysis of protein–protein and other biomolecular interactions.

## Figures and Tables

**Table 1:** The approximate time (hours) of saturation for allergens-sIgE complexes for hydrogel or brush biochips or brush biochips with mixing.

Type of Biochip		Hydrogel Biochip		Brush Biochip		Brush Biochip + Mixing	
	Parameter	Parameter B ^a^	Time of Saturation, x_sat_^b^	Parameter B	Time of Saturation, x_sat_	Parameter B	Time of Saturation, x_sat_
Allergen or Antibody							
D1		4 ± 2	18	0.3 ± 0.05	1	0.4 ± 0.2	2
E1		7 ± 1	24	2.0 ± 0.6	8	1.5 ± 0.9	7
E5		11 ± 3	42	0.3 ± 0.2	2	0.6 ± 0.2	2
M6		–	–	0.5 ± 0.3	2	0.6 ± 0.2	2
T2		10 ± 2	36	1.6 ± 0.2	5	0.8 ± 0.2	3
T3		6 ± 1	21	1.7 ± 0.2	6	1.0 ± 0.2	4
T4		0.9 ± 0.9	5	1.8 ± 0.3	6	1.1 ± 0.3	4
T14		4 ± 1	15	1.7 ± 0.2	6	0.9 ± 0.2	3
W1		–	–	0.6 ± 0.5	3	0.6 ± 0.2	2
W6		0.9 ± 0.9	5	1.0 ± 0.3	4	0.5 ± 0.2	2
W15		–	–	1.0 ± 0.3	4	0.6 ± 0.2	2
G3		9 ± 1	30	0.8 ± 0.3	3	0.6 ± 0.2	2
G4		7 ± 1	24	0.8 ± 0.1	3	0.8 ± 0.2	3
G5		7 ± 1	24	0.7 ± 0.2	3	0.7 ± 0.1	2
G6		8 ± 2	30	0.8 ± 0.2	3	0.9 ± 0.3	4
G8		5 ± 1	18	0.7 ± 0.5	4	0.7 ± 0.2	3
G13		6 ± 1	21	2.7 ± 1.0	11	0.4 ± 0.2	2
Bet V1		–	–	0.04 ± 0.04	0.2	0.6 ± 0.2	2
Bet V2		–	–	0.5 ± 0.4	3	0.7 ± 0.2	3
Bet V4		–	–	0.5 ± 0.3	2	0.7 ± 0.2	3
GoatAHIgE		2 ± 1	9	0.4 ± 0.3	2	0.7 ± 0.2	3
Averaged time of saturation		22		4		3	

^a^ Parameter B was calculated during approximation of experimental points using equation. y = A × (1 − exp(−x/B)) (see also legend to Figure 1). ^b^ Time of saturation, x_sat_, was calculated as x_sat_ = −ln(0.05) × (B + ΔB), where ΔB is the standard error.

## Data Availability

Data is contained within the article.
